# (Dis)respect and shame in the context of ‘medically unexplained’ illness

**DOI:** 10.1111/jep.13740

**Published:** 2022-07-27

**Authors:** Katharine Cheston

**Affiliations:** ^1^ Institute for Medical Humanities Caedmon Building Durham UK

**Keywords:** disrespect, medically unexplained, medically unexplained symptoms, respect, shame

## Abstract

A significant proportion of somatic symptoms remain, at present, medically unexplained. These symptoms are common, can affect any part of the body, and can result in a wide range of outcomes—from a minor, transient inconvenience to severe, chronic disability—but medical testing reveals no observable pathology. This paper explores two first‐person accounts of so‐called ‘medically unexplained’ illness: one that is published in a memoir, and the other produced during a semi‐structured interview. Both texts are revelatory for their expression of shame in the context of encountering disrespect from healthcare professionals. The first section of my paper, clinical encounters, explores disrespect which, I argue, takes three interconnecting forms in these texts: disrespect for pain when it is seen as ‘medically unexplained’, disrespect for the patient's account of her own pain, and disrespect for the patient herself. The second section elucidates the shame that occurs as an affective and embodied consequence of encountering such disrespect. I claim that patients living with so‐called ‘medically unexplained’ illnesses suffer a double burden. They endure both somatic and social suffering—not only their symptoms, but also disrespectful, traumatic and shame‐inducing experiences of healthcare systems. I conclude with a reflection on the urgent need for changes in clinical training that could improve the quality of life for these patients, even in the absence of an explanation, treatment or cure for their symptoms.

## 1 INTRODUCTION

A significant proportion of somatic symptoms remain, at present, medically unexplained.^1^, ^2^, ^3^, ^4^, ^5^ These symptoms can affect any part of the body, resulting in a wide range of outcomes—from a minor, transient inconvenience to severe, chronic disability—but medical testing reveals no observable pathology, no known disease mechanism.^2^, ^3^, ^4^, ^5^ Such medically unexplained symptoms can occur in isolation or as syndromes comprising multiple, chronic and (often) disabling symptoms.^6^, ^7^, ^8^ While rates of prevalence vary, it is thought that these symptoms represent one of the largest categories of complaints in primary care.^1^, ^5^, ^7^, ^9^, ^10^ Women are up to three times more likely to be affected than men.^2^, ^6^, ^11^, ^12^, ^13^

These phenomena are discussed in both the clinic and the literature under a variety of diagnostic labels, including ‘Medically Unexplained (Physical) Symptoms’, ‘Persistent Physical Symptoms’, ‘Complex Physical Symptoms’, ‘functional symptoms’, ‘illness without disease’ and ‘symptoms that cannot be classified’.^14^, ^15^, ^16^, ^17^, ^18^ Such variety is indicative of the disagreement that plagues this field. For example, many patients with ‘medically unexplained’ illnesses strongly believe that an organic cause for their symptoms will eventually be discovered, given time and research funds.^19^, ^20^, ^21^ However, this is at odds with many clinicians—such as neurologist Suzanne O'Sullivan—who believe equally strongly that, in the majority of these cases, ‘no disease is found because there is no disease to find’; if symptoms cannot be medically explained, then the cause is most likely to be psychological or behavioural.^22^

Whilst this debate rages on, there has been a recent boom in publications of memoirs providing first‐person accounts of so‐called ‘medically unexplained’ illnesses.^23^ One such example is Sarah Ramey's *The Lady's Handbook for Her Mysterious Illness: A Memoir*.^24^ Ramey lives with debilitating physical symptoms, including excruciating pain and severe gastrointestinal issues, but—for many years, and for most of her memoir—her illness remains, as she terms it, ‘mysterious’. As a result, she encounters stigmatization and a lack of support; her memoir, like so many others written about these ‘mysterious’ conditions, details not only somatic but *social* suffering. Towards the end of her memoir, Ramey speaks directly to her reader, posing an important question:

I just wanted people around me to treat me the way they would treat any other sick person they knew—with dignity, and care, and respect.

Why was that too much to ask?^24^

Here, Ramey appears to set up a distinction between ‘other sick [people]’, who are treated ‘with dignity, and care and respect’—and those living with ‘mysterious’ or ‘medically unexplained’ conditions who, by implication, are treated with neither dignity, nor care nor respect.

This paper will analyse two contrasting accounts of ‘medically unexplained’ illnesses to interrogate Ramey's question: why is respectful treatment ‘too much to ask’ for these patients? This analysis will also explore the affective consequences of encountering disrespect in clinical encounters, with a particular focus on shame. Finally, it will consider the implications this may have for clinical practice.

## 2 METHODS

The first of these accounts is Ramey's aforementioned memoir, which tells—over 400 pages of finely considered prose—an emotive account of her experiences of seeking healthcare, in the United States, for a constellation of ‘mysterious’ symptoms.^24^ The second account is told by a woman who we will call Arya. Arya is a British Asian Indian woman, in her 30s, living in the United Kingdom. Like Ramey, Arya lives with gynaecological and gastro intestinal symptoms for which healthcare professionals cannot find a structural cause. I interviewed Arya in the summer of 2021: this was a semistructured interview, lasting 1.5 h, which was conducted using videoconferencing software. My analysis of Arya's account draws from a transcript produced verbatim from an audio recording of our interview, as well as from my field notes completed at the time of the interview.

These two accounts are drawn from a larger project, which analyses 10 memoirs alongside 10 interview transcripts and aims to understand, theorize and, ultimately, propose means of alleviating the shame experienced by women whose symptoms are seen to be ‘medically unexplained’. Ramey's memoir and Arya's spoken testimony are particularly revelatory for their expression of shame in the context of disrespect. This paper offers a focused analysis of these two very different first‐person accounts so as to probe the connections between these two affective, embodied experiences. The choice to bring literary text(s) and interview transcript(s) into dialogue was made so as to draw attention to the expression of experiences which can be hard to talk or write about. Through an innovative blend of thematic and literary analysis that attends to the language, imagery, themes, form and structure of these very different first‐person accounts, I aim to reveal aspects of these hidden experiences that might otherwise remain unseen or unnoticed.

Respect and shame both have long critical histories, and vibrant inter‐ and multidisciplinary communities continue to discuss and conceptualize their importance in relation to healthcare.^25^, ^26^, ^27^, ^28^, ^29^, ^30^, ^31^, ^32^, ^33^, ^34^, ^35^ In this paper, I build from recent work in bioethics that advocates for the relevance of the concept of ‘respect for persons’ to experiences of healthcare, and I adopt the definition of respect developed by Beach et al.: ‘respect as recognition of the unconditional value of patients as persons […] irrespective of a patient's personal characteristics’.^25^, ^26^, ^27^, ^36^, ^37^, ^38^ However, my primary focus in this paper is experiential, not theoretical. I aim to explore respect, in Subramani and Biller‐Andorno's words, ‘as an embodied concept’: respect as it is experienced (in its presence or absence) and expressed in two first‐person accounts.^25^

I am especially interested in the potential links between (dis)respect and shame in these ‘medically unexplained’ contexts. I take shame to be a negative self‐conscious emotion that results from feeling seen (whether the other is actually present or merely imagined) to be faulty, inadequate or worthless. My understanding of shame is informed by recent work on the phenomenology of shame in medicine, which has revealed shame to be a potent, pervasive force in clinical encounters.^33^, ^34^, ^35^ Taking the two aforementioned texts as case studies, this paper explores how shame can be an embodied consequence of encountering disrespect in healthcare settings. Moreover, it elucidates the unique affective and embodied features of this sense of shame that those with ‘medically unexplained’ illness may be especially vulnerable to experience.

My analysis of these texts will be divided into two sections. The first section, clinical encounters, elucidates the disrespect Arya and Ramey experience while seeking healthcare for their ‘medically unexplained’ symptoms. The second section focuses on shame as an affective consequence of this disrespect. Finally, I conclude with some reflections on improvements that could be made to clinical practice and training in this regard.

## 3 CLINICAL ENCOUNTERS

Both Ramey and Arya describe a sudden onset of severe symptoms, which prompt them to contact healthcare professionals. Arya's pain appears unexpectedly: ‘I literally, what seemed like out the blue, started getting these sharp shooting pains in my clitoris’, she recounted. The pain—which ‘was kind of like random, sharp, nerve like shooting pains’—was severe enough to prevent her from working and to prompt her to seek immediate help from her GP:

I was like, what is this? Like you know is this some kind of bizarre urine infection? Like what is this? So I did all of the right things, like contacting the GP and I got completely fobbed off, like they were all like it's nothing, like just ignore it, like it's nothing kind of thing.

In contacting her GP, Arya did what she might reasonably have been expected to do when experiencing distressing and confusing symptoms; she did, as she stresses, ‘all the right things’. Arya's GP (and later, GPs), by implication, didn't do *all the right things* as she was, in her own words, ‘completely fobbed off’. Over the course of at least five GP appointments, Arya seems to have felt distinctly and repeatedly dismissed: ‘they were all like it's nothing, like just ignore it, like it's nothing kind of thing’. In desperation, Arya pays a significant amount of money for a private consultation with a gynaecologist—but, she says, ‘again, they were like oh it's nothing’. Arya does not go into much detail as to the impact of these appointments, but her gestures and tone of voice are revelatory: she imitates her doctors speaking to her as if brushing her complaints away and—my field notes record—her hand gestures enact this brushing movement. It's apparent she considered this treatment to be dismissive, belittling: she is fobbed off, brushed away and her pain is reduced, as she says, to ‘nothing’.

Arya's experiences of secondary care are no more positive: ‘the first gynaecologist appointment I had’, she told me, ‘the woman was like there's nothing wrong with you, are you sure you're not allergic to cheese or chocolate’. This particular appointment seems a memorable one for Arya, who returned later in the interview to expand upon her previous comments:

the gynae that I told you about who said oh it might be cheese or chocolate that you're allergic to, […] she was like there's nothing wrong with you, just go home, use more lube during sex basically. And I was like, I'm not having fucking sex, like I wouldn't be using … she was like, oh use more lube you'll be fine kind of thing.

In each of the two times that Arya discusses this particular appointment, she attributes the same phrase to this gynaecologist: ‘there's nothing wrong with you’. This phrase has echoes of her GPs’ and private consultant's comments—*oh it's nothing*—but appears all the more distressing: it seems like a more explicit rejection of her pain and its impact. In response to her gynaecologist's advice to ‘just go home, use more lube during sex’, Arya appears to try to express the limitations her pain imposes on her, saying, ‘And I was like, I'm not having fucking sex, like I wouldn't be using …’ The impact of her pain on her relationship with her partner was, perhaps, too painful or too personal to discuss in detail during the interview, as it disappears into the pause marked by the ellipses. However, Arya's expletive shines some light on the frustration she felt in this appointment, while the return of the dismissive phrasing attributed to the gynaecologist (‘*oh* use more lube’) suggests that she was made to feel not only as if her pain didn't matter, but also as if her suffering as a result of this pain didn't matter either.

Ramey uses similar language as she sums up her experiences with healthcare professionals: ‘Despite my truckload of symptoms, it appeared I had nothing at all’.^24^ Like Arya, Ramey employs the word ‘nothing’: with medical testing and examinations revealing nothing significant, it would seem that those with ‘medically unexplained’ or ‘mysterious’ illnesses are made to feel their symptoms *are* nothing, insignificant. Ramey expands upon this:

Again and again and again (but not without a stop at the billing counter), doctor after doctor reached a hand inside of me, rooted around, caused me extraordinary, blinding pain […]. If they couldn't find a diagnosis, or a lump, or something tangible […] instead of saying, ‘Well, we don't know, but let's keep trying,’ they all began to recommend psychiatric counselling.

Again. And again. And again.

[…] Mostly, it just didn't stand to reason. Even if no one believed me about the aching and the itching and the pelvic pain—which, I get it, you can't see on a scan or under the microscope—there was still hard evidence of something wrong. The fevers. The soccer‐ball stomach. The swollen labia. The furry tongue.

It didn't matter.^24^

In just one short, stand‐alone sentence—‘It didn't matter’.—Ramey implies a bleak, helpless resignation in the face of repeated disconfirmation of her subjective experience. Interestingly, it is not just Ramey's narrative of subjective symptoms that ‘didn't matter’; ‘hard evidence’, too, is rejected when it does not correspond to ‘a diagnosis, or a lump, or something tangible’. Ramey makes the most of the expansive, expressive context of the literary memoir, employing repetition—‘Again and again and again […] doctor after doctor […] Again. And again. And again.’—to reiterate that her clinical encounters were characterized, as standard, by disbelief and dismissal.

Similar, yet subtler, repetition is also present in Arya's spoken account. For example, she attributes the same language (‘oh it's nothing’) to her GPs, her private consultant, and the NHS gynaecologist. Moreover, at times her speech moves almost imperceptibly from the singular to the plural, such as ‘I did all of the right things, like contacting the GP and I got completely fobbed off, like they were all like it's nothing’. Both Arya and Ramey implicate the whole healthcare system in their mistreatment. They do not speak of one unusually bad experience with one particular healthcare professional; bad experiences are, for them, the norm. This begs the question: can patient experience only be respected—and not dismissed, belittled or disbelieved—when it is corroborated by the results of medical testing and imaging that healthcare professionals currently have at their disposal?

While Arya and Ramey are, eventually, successful in obtaining a diagnosis, this does little to alter the disrespect they encounter in clinical spaces. For example, Arya's diagnosis of vulvodynia (vulvar pain of unknown aetiology) comes at a cost, as she describes the diagnostic process as *traumatizing*:

actually the getting the diagnosis is traumatising in its own. It's ‘cos it's like you're not believed I think… I think it's almost like every time I was treated like I was making up what I was saying, like I'm just this person that's over‐exaggerating, when actually I'm the opposite, I underplay what's wrong with me. So… so yeah, I think the way the […] medical system went about dealing with me was completely like all the wrong things to do almost.

What I find particularly noteworthy about this passage is the aspect of this experience that Arya describes as traumatic: it is the fact that she was disbelieved, that she was (repeatedly) ‘treated like [she] was making up what [she] was saying’. The examples I have cited from Arya's interview and Ramey's memoir have provided evidence of disrespect for patients’ accounts of their own illnesses when these do not correspond with the results of objective medical testing. What Arya is describing here, however, goes further than this. She seems to be expressing experiences of more generalized disrespect—disrespect directed at her as a person. She describes being misunderstood and misrepresented: being treated ‘like I'm just this person that's over‐exaggerating’. Here her use of the adverb ‘just’ implies that she may have felt belittled, or even ridiculed. It is not only her pain that is trivialized; she felt personally invalidated by these experiences. This appears to be a deeply distressing experience for Arya, who took care to correct this misreading of her personality in the interview. The implication seems to be that, in the absence of an objective explanation for her symptoms, it is Arya herself who is at fault; if there was *nothing wrong* with her, from a biomedical perspective, it must be Arya herself—her personality and behaviour—that is wrong.

The disrespect that Arya and Ramey encounter in clinical encounters is multifaceted; it creates distinct layers of distress. Firstly, their pain itself is dismissed when it is not confirmed by objective evidence; secondly, their accounts of their experiences are minimized, or even rejected; and, thirdly, they are met with disrespect for themselves as people. This is most apparent in Ramey's extended discussion of her encounter with a ‘renowned urologic surgeon’, which brings Arya's description of the diagnostic process as ‘traumatising’ into a new light.^24^ Ramey travels cross‐country to attend an appointment with this surgeon—only to find that, ‘like everyone else, he had not known what to make of my case and had referred me down the line’.^24^ A few days after her initial appointment, however, Ramey contacts his office to discuss her ‘extremely swollen, bright red left labia’.^24^ She speaks to a junior on‐call physician, who agrees that the next step should be to conduct a labial biopsy under general anaesthesia.

When Ramey arrives for her scheduled appointment with the urologic surgeon, she is shocked to find that he was not at all happy to see her:

now that I had returned after he had already said he didn't know what to do about my case, he seemed to have transformed from a once‐dispassionate physician of some renown, Dr. Jekyll if you will, to a very different man—a urogynocologic Mr. Hyde.^24^

‘[V]ery begrudgingly’, Ramey writes, this ‘urogynocologic Mr. Hyde’, agrees to examine her.^24^ When she asks him what she should do about her swollen labia, the surgeon retorts ‘I would stop worrying about it so much’.^24^ Reluctantly, the surgeon agrees to do the biopsy—but only using local anaesthesia. Ramey tries to insist on the general, ‘[b]ut Hyde fixed [her] with a look of such naked disgust, [she] felt afraid’.^24^ Looking up at the ‘large, ticking clock on the wall’, Hyde tells Ramey that he doesn't have time to take her to the operating room ‘for something so trivial’.^24^ Ramey is given two choices: either ‘toughen up and have the biopsy done right there with a local anaesthetic’ or leave.^24^ Ramey's severe pain is trivialised but, feeling vulnerable—she's partially clothed, her feet in stirrups—she decides she ‘needed to toughen up’ and agrees to the procedure.^24^

What follows makes for difficult reading. Hyde prepares a ‘long needle filled with lidocaine’ and inserts it into Ramey's ‘bright red, swollen left labia minora’.^24^ Ramey screams ‘so loud, the nurse had to hold [her] shoulders down’.^24^ Ramey waits for 10 min, as instructed, for the lidocaine to take effect. After the allotted time has passed, the area is still not numb—much to the surgeon's displeasure:

Sighing, he inserted the long needle into my bright red, swollen left labia minora again, and injected the lidocaine again.

I was screaming as if my own child were being murdered.

[…] He poked it again, and I could still very much feel the touch and the pain […] but he declared that just wasn't possible, that there was no way I could feel anything anymore, told me to be quiet, and proceeded to start cutting into my labia.

I have never cried so hard in my life.

And throughout the procedure, Mr. Hyde would not look me in the eye.

He scooped three oozing red samples onto three glass plates for the laboratory.

Then without saying a word, he cauterised the wound.

And when he was finally done, he simply walked out of the room.^24^

Ramey's screams are met with exasperated, disinterested silence; the surgeon denies her pain outright, declaring that it ‘just wasn't possible’. We might imagine that it would be difficult to find a more flagrant example of the disrespect that patients with ‘medically unexplained’ health conditions encounter from healthcare professionals. Ramey's account of her own pain, even when expressed in such a visceral, primal form, is completely dismissed by this surgeon—who doesn't even offer her the simple courtesy of looking her in the eye.

All the more distressing, however, are the more subtle forms of disrespect that Ramey encounters in this appointment. After the biopsy, the nurse asks Ramey which pain medication she would prefer. Ramey declines—she doesn't, she explains, tolerate pain medication well. The nurse is insistent, and eventually, Ramey agrees to take a single dose of Percocet (an opioid medication, containing oxycodone and paracetamol). However, the nurse returns with an ice pack, but no medication. She reports, Ramey writes, ‘eyes fixed firmly on her shoes, that the surgeon had refused to prescribe pain medication for me. […] Eyes on shoes, she said that this particular surgeon didn't like to give pain medication to “patients like you”’.^24^ When Ramey asks as to the reasons for this refusal, the nurse explains that ‘patients with chronic pain can often go to… great lengths to obtain opioids’.^24^ The impact of these comments appears, if possible, even more agonizing for Ramey than her surgeon's scalpel:

I realised that I was trembling and tears were falling down my face. I could not think of a single word to say. All I could think about was what kind of person you would have to be to travel long distances for a vaginal biopsy and cauterisation just to score one tablet of Percocet—and that my doctor thought I was that person. I took the ice pack in silence, put it wincingly between my legs, sank into the wheelchair, and turned my face away.^24^

Ramey's screams are replaced by quiet tears; she is reduced, finally, to silence. As she turns her face away, Ramey appears to admit defeat, overwhelmed by her doctor's misjudgement of her and her motivations. The tears falling down her face are not the result of the physical pain of the botched biopsy, but the emotional distress upon encountering such profound and painful disrespect—for her experience, for her pain and for herself.

## 4 CONSEQUENCES

Confronted with the limits of his own knowledge—embodied by Ramey and her inexplicably ‘bright red, swollen left labia minora’—Ramey's surgeon responds irrationally and illogically. His palpable rage appears driven by shame, and indeed he fulfils each of Lazare's clues indicating a shame response in the physician: anger at the patient, inadvertent humiliation of the patient, and the wish not to see the patient again.^32^ Further evidence is the surgeon's avoidant behaviour, as he evades eye contact and escapes from the situation: not only does he leave the room immediately after the procedure, but he actually leaves the building entirely, requesting no further contact related to Ramey's case.^24^ The nurse, too, exhibits a shame response, speaking with ‘eyes fixed firmly on her shoes’.^24^ However, while shame pervades this encounter, it is Ramey who bears its full impact. Lazare argues that ‘[a]ngry responses’ to shame include displacing shame ‘onto someone lower in the pecking order’, so it is no surprise that Ramey—the most vulnerable, and least powerful, individual in this encounter—is, ultimately, left sinking into a wheelchair, turning her face away.^32^

While this episode is undoubtedly an extreme example, it speaks of a widespread issue encountered by those with ‘medically unexplained’ illnesses. Lazare writes of ‘physicians who are particularly shame‐prone over their need to see themselves as perfect and in complete control in their practice of medicine’.^32^ By their very definition, ‘medically *unexplained’* illnesses challenge this sense of perfection, control and certainty, which might render physicians—as well as patients—particularly prone to experience shame in consultations regarding these symptoms. Ramey's and Arya's accounts reveal that, instead of acknowledging this shame—and accepting that medical knowledge is, and may always be, incomplete—physicians can discharge their shame onto the patient, who is made to feel that she, and not the limits of medicine, is the problem.

For example, Arya spoke in her interview about how, as a society, ‘we are conditioned with Western medicine being like this superior thing […] we place [doctors] as this like superior being of knowledge’. ‘But’, she counters, ‘I think there's some things that they just don't understand’. Arya senses that her doctors’ unwillingness—or perhaps inability—to admit the limits of their knowledge may have contributed to their inability to acknowledge her pain compassionately. Instead, she reflects, they ‘just sort of fixated on’ her behaviour ‘as the source of the problem’. This has parallels in the following passage from Ramey's memoir:

every single time the heroes did more damage, every single time people continued to treat me in ways that were hurtful and demeaning […]—reader, I could not stop myself from boomeranging back to the message I had been programmed with subtly and unsubtly at every single step of the way:

The problem was me.^24^

Here, the crated rhetoric of the literary text, with its repeated clauses, allows the pain of this self‐blame to ring out—‘every single time’. As they continue to be treated in ways that are ‘hurtful and demeaning’, both Arya and Ramey are forced to come to the same conclusion: the blame is theirs alone.

For both Arya and Ramey, this provokes a deep sense of shame—shame that is not only acute and intense (as Ramey displays after her encounter with the surgeon), but is also chronic, corrosive and characterized by self‐blame and self‐doubt. ‘It sort of feels like it's your own fault somehow’, Arya admits, ‘that you're like in this situation sort of thing. And because the… [medical] system are acting so normal about it and dismissing you, you're left with nothing but yourself’. For Arya, this sense of blame triggers an intensely distressing sense of shame, as she discloses: ‘I did feel ashamed, not must have, I did, definitely did, when I had all those back‐to‐back conversations with the GP, when I went to the gynaecologist’. Arya elaborates as to the particular nature of this shame experience: ‘I was just like oh gosh, you know, this is really bad, like you know why did this happen to me, like you know maybe I did something wrong, like maybe I worked too hard or I don't know, I don't know what it was’. Arya's distress is evident in the rapid pace of her speech, as she discloses—with apparent difficulty—painful feelings of shame, intermingled with self‐blame and self‐doubt.

Ramey's experiences of shame appear to echo Arya's. She writes frankly about shame, making clear that ‘shame is a big issue’ for women with ‘mysterious’ illnesses—who are, by her own definition, ‘addled, embarrassed, ashamed, and inflamed’.^24^ Ramey's memoir suggests, in a way remarkably similar to Arya's interview testimony, that the particular nature of the shame that women with ‘mysterious’ illnesses experience is chronic, characterized by self‐blame and self‐doubt. This is made most evident in the following episode, which occurs after Ramey acknowledges that she is ‘out of options’ for solving her ‘mysterious’ illness:

This must be what I want, I conceded. *I accept I may have created my own illness*, I wrote on a sticky note and attached it to my mirror. *Take responsibility*, I wrote on another. […] I decided I just needed to accept that I was the attention‐seeking malingerer those emotional healers had hinted at […] I didn't have a parasite—I was the parasite. […]

*I am a monster*, I thought.^24^

Ramey is left, just like Arya, with *nothing but herself* and, in her anguish and despair, comes to a tragic conclusion. The extreme nature of her self‐blaming and self‐shaming—expressed in italics, which mimic her inner voice—is shocking, and indeed distressing. Ramey is sunken in shame: sobbing, curled up in the foetal position, her mind racing ‘between which wrong food I had eaten, which wrong emotion I was feeling […]—and on and on and on’.^24^ This has a corrosive, destructive impact on her self‐esteem and self‐worth, evident as she declares to her readers: ‘I was a failure’, ‘I was disintegrating’, ‘I was destroyed’.^24^ Written from within what Ramey terms her ‘isolation shame‐ber’, her unflinching prose permits her readers to witness her chronic, corrosive shame, tinged as it is with expressions of self‐blame and self‐doubt—laying bare on the page all that she might find too painful to reveal in conversation.^24^

## 5 CONCLUSIONS

Patients with so‐called ‘medically unexplained’ illnesses suffer a double burden; they endure both physical suffering and traumatic experiences in healthcare systems. As I have explored, the disrespect that Ramey, Arya and others encounter from healthcare professionals takes three interconnecting forms: disrespect for pain when it is seen as ‘medically unexplained’, disrespect for the patient's account of her own pain, and disrespect for the patient herself. In some instances, such as Ramey's encounter with the urologic surgeon, this disrespect might betray a shameful response in the physician.^32^ Indeed, ‘medically unexplained’ illness, which highlights the limits of medical knowledge and power, might be a particularly shame‐inducing experience for both patient and physician. Arya's and Ramey's accounts reveal that shame can be displaced onto the patient, who is made to feel that her illness is her own fault. This results in a profound and distressing sense of shame, which is chronic, provokes intense feelings of self‐blame and is seen to be uniquely corrosive to self‐worth.

This has significant implications for clinical practice. While flagrant disrespect—such as that which Ramey experienced at the hands of her urologic surgeon—is obviously an example of poor practice, these textual case studies have illustrated that, for those with ‘medically unexplained’ illness, disrespect pervades clinical encounters in subtle yet shame‐inducing ways, many of which clinicians may be utterly unaware. This highlights the desperate need to reconsider clinical training related to ‘medically unexplained’ conditions. To take UK medical education as an example, ‘medically unexplained’ illnesses are said to receive little attention during undergraduate medical training and, indeed, they are often entirely absent from curricula.^39^, ^40^, ^41^, ^42^ Considering how common ‘medically unexplained’ conditions are—and that they may pose a unique affective challenge to clinicians—to exclude them from medical curricula surely benefits neither clinicians nor their patients.

More training is urgently needed in this area, from the earliest days of undergraduate education, through to qualification and beyond. Crucially, this training must focus on patients’ lived experiences, as well as on empowering physicians. Firstly, as the literature shows that any training that undergraduate medics do receive on ‘medically unexplained’ illnesses can be influenced by their tutors’ negative perceptions of these conditions, it is imperative that this training is co‐developed and co‐delivered together with those with lived experience.^39^ Sharing first‐person accounts can be a powerful tool in medical education—as, I believe, Ramey's and Arya's accounts illustrate.^43^, ^44^, ^45^, ^46^ Secondly, this training needs to empower clinicians and enable them to approach these ‘medically unexplained’ encounters with confidence, with the aim of shielding them from the shame that may be the natural consequence of encountering the limits of medical knowledge. I hope that this paper and these case studies have shown that physicians still possess immense power to improve their patients’ quality of life—through respectful, patient‐centred, shame‐sensitive treatment.

## CONFLICT OF INTEREST

The authors declare no conflict of interest.

## Data Availability

Due to the nature of this research, participants of this study did not agree for their data to be shared publicly, so supporting data is not available.
